# Evaluation of the Functional and Radiological Outcomes of Fixed Angle versus Variable Angle Volar Locking Compression Plates in Managing Intra-articular Fractures of Distal End Radius

**DOI:** 10.5704/MOJ.2507.002

**Published:** 2025-07

**Authors:** DK Garg, H Sakale, AC Agrawal, B Kar, E Pandiyarajan, SK Yadav

**Affiliations:** 1 Department of Orthopaedics, All India Institute of Medical Sciences, Bathinda, India; 2 Department of Orthopaedics, All India Institute of Medical Sciences, Raipur, India

**Keywords:** distal radius fracture, variable angle plate, fixed angle plate, mayo wrist score, radiological outcomes

## Abstract

**Introduction:**

Distal end radius fractures are common upper extremity fractures often requiring surgical intervention for instability. This study compares the functional and radiological outcomes of fixed angle versus variable angle volar locking compression plates in managing these fractures.

**Materials and Methods:**

A prospective randomized study was conducted at AIIMS, Raipur, from July 2020 to December 2022. Patients aged 18-60 years with complete intra-articular distal end radius fractures (AO Type 23C1, 23C2, 23C3) were included. Sixty-four patients were randomized into two groups: one receiving fixed angle plates (Group 1) and the other variable angle plates (Group 2). Functional outcomes were assessed using the Mayo wrist score, and radiological outcomes were evaluated with the Sarmiento modification of Lindstrom’s criteria. Statistical analysis was performed using IBM SPSS 22.0, with p-values <0.05 considered significant.

**Results:**

At 3 months, the variable angle group had significantly higher Mayo wrist scores (63.12 ± 11.81) compared to the fixed angle group (48.75 ± 11.90, p=0.005). This trend continued at 6 months (75.93 ± 9.16 vs. 64.37 ± 14.59, p=0.025) and 1 year (91.87 ± 7.27 vs. 81.25 ± 16.17, p=0.044). Radiologically, the variable angle group demonstrated better volar tilt restoration at all follow-up points (p<0.001 at 3 months, p=0.001 at 6 months, p=0.004 at 1 year). Complication rates were similar between groups.

**Conclusion:**

Variable angle volar locking compression plates offer superior functional outcomes and better volar tilt restoration compared to fixed angle plates for unstable distal end radius fractures. Both types exhibited similar complication rates.

## Introduction

Distal end radius fractures are among the most common fractures of the upper extremity^1^. Most of these fractures can be managed conservatively, while some unstable fractures require surgical intervention. Various treatment modalities are available for the fixation of distal radius intra-articular fractures, such as closed reduction and cast application, percutaneous K-wire fixation, external fixation, and open reduction and internal fixation with plating^2^.

In the younger age group, patients have high demands regarding functional outcomes. This age group mainly experiences intra-articular and comminuted fracture patterns due to high-velocity trauma, which limits the role of conservative management. Open reduction and internal fixation with plating are common methods to treat intra-articular fractures^3^. This approach provides anatomical reduction and stable fixation of fracture parts, which helps restore the functional outcome of the patient and aids in early mobilization and return to work^4^.

Certain fracture patterns are difficult to fix with conventional plates, such as comminuted fractures and fractures that cross the watershed line. Conventional plates have greater thickness, which can cause prominence, tendon irritation, and poor penetration of fracture parts that cross the watershed line^5^. Variable angle plates are biomechanically comparable to fixed angle plates^6^. Recently, low contour plates have become available, offering less skin prominence and reduced tendon irritation^7^. Variable angle plates allow for a 15° variation, which aids in better fixation of comminuted parts, the radial styloid process, the lunate facet, and die-punch fracture patterns^8^.

The purpose of our study was to compare the functional and radiological outcomes of unstable distal end radius fractures treated with fixed-angle and variable-angle volar locking compression plates.

## Materials and Methods

This is a prospective randomized study conducted at the Tertiary Health Care Center, AIIMS Raipur Orthopaedics Department, after receiving approval from the Institute's Ethics Committee. The study was conducted between July 2020 and December 2022. All patients who presented to the trauma and emergency department and the outpatient department (OPD) of the Orthopaedics Department at AIIMS Raipur with intra-articular distal end radius fractures were included in the study after matching the inclusion and exclusion criteria. Patients aged between 18 and 60 years with complete intra-articular fractures (AO Type 23C1, 23C2, 23C3) and willing to participate in the study were included. Exclusion criteria were open fractures, ipsilateral limb fractures other than the distal end radius, extra-articular and partial intra-articular fractures, and unwillingness to participate in the study.

**Fig. 1:**
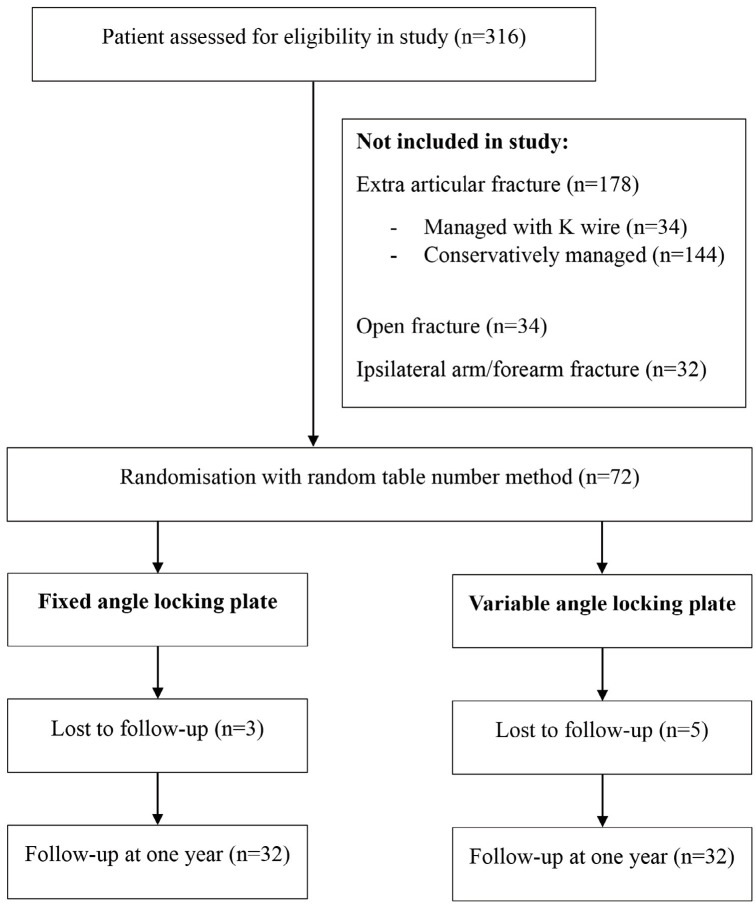
Flowchart of inclusion and exclusion of patient in the study.

After informed consent, eligible patients who were willing to participate in the study were randomized using a computer-generated random table number method into Group A (Patients with intra-articular Fractures distal end radius treated using a Fixed Angle Locking Compression Plate) and Group B (Patients with Intra articular Fractures distal end radius treated using Variable Angle Locking Compression Plate) (Fig. 1).

**Fig. 2:**
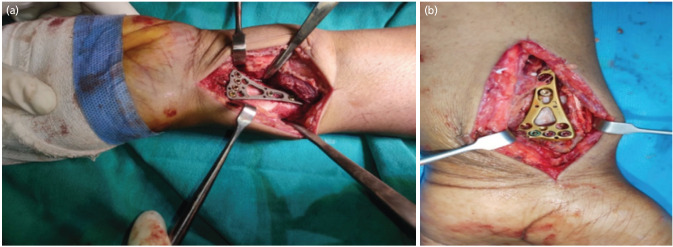
Intra-operative procedure showing the various plates using in the study. (a) fixed angle plate; (b) variable angle plate.

All patients were operated on using the Modified Henry approach. Pre-operative and post-operative care were provided according to the standard treatment protocol. The implants used were two columns of distal radius plate 2.4, VA-LCP (Synthes), and locking compression plate 2.4, FA-LCP (Synthes). The variable angle LCP allows for a 15° deviation for the locking of screws, while the FA-LCP has a fixed trajectory with a predetermined angle. There is a difference in the shape of the head of the screw between the fixed angle and variable angle locking compression plates. The cone-shaped head of the fixed angle locking screws allows fixation only orthogonal to the plate hole, whereas the cup-shaped head of the variable angle locking screw allows a 15° deviation (Fig. 2).

**Fig. 3:**
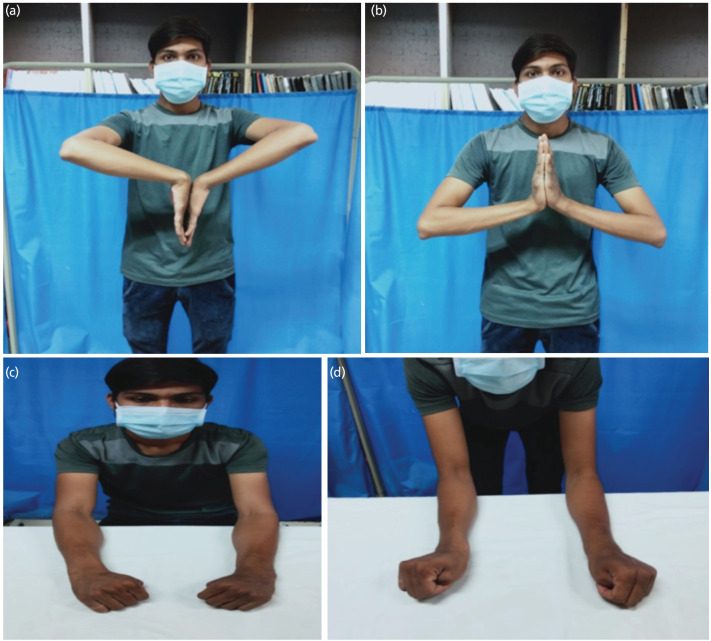
Functional Outcome among patient. (a and b) Range of motion (Wrist flexion and extension). (c and d) Deviation (Radial and

Post-operative follow-up was done at three months, six months, and one year. Functional outcomes were evaluated with the Mayo wrist score, and radiological outcomes were evaluated using the Sarmiento modification of Lindstrom criteria. Functional outcome evaluation was done using the Mayo wrist score, which has four components: pain, satisfaction, range of motion, and grip strength. Each component has a maximum score of 25, making the total score out of 100 (Fig. 3)^9^.

**Fig. 4:**
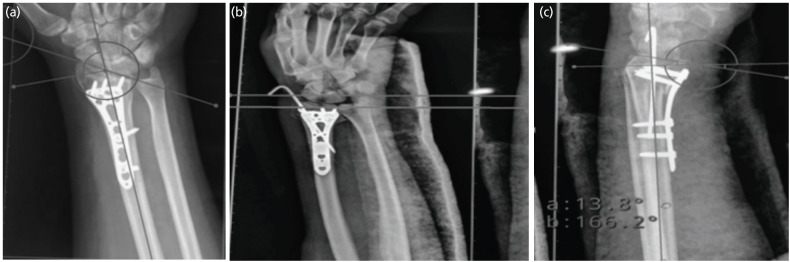
Radiological outcome. (a) Radial Inclination. (b) Radial Shortening. (c) Volar Tilt.

The radiological outcome was evaluated using the Sarmiento modification of Lindstrom’s criteria, measuring radial height, volar tilt, and radial inclination on post-operative radiographs with the help of the Angulus app (Fig. 4)^10^.

Statistical analysis was carried out using IBM SPSS 22.0 for Windows [SPSS Inc., Chicago, IL, USA]. Continuous and categorical variables were expressed as mean ± SD and frequency (%), respectively. Descriptive statistics were done for the distribution of age, gender, and affected side. A paired t-test was used to compare the mean total score and components of the score between Group 1 and Group 2 at one month, three months, and six months. A Chi-square test was used to compare the functional and radiological outcome (grading) of Group 1 and Group 2 at one month, three months, and six months.

## Results

The study included 64 patients, evenly divided between those treated with fixed angle LCP (n=32) and variable angle LCP (n=32). The mean age was 38.81±9.60 years in the fixed angle group and 34.13±7.46 years in the variable angle group (p=0.120). Both groups had identical sex distributions, with 87.5% male and 12.5% female (p=1.000). The mechanism of injury differed significantly (p=0.039), with 87.5% of fixed angle cases due to road traffic accidents (RTA) and 12.5% due to falls on outstretched hands (FOOSH), while 100% of variable angle cases were due to RTA. The dominant hand was affected in 37.5% of the fixed angle group and 25.0% of the variable angle group (p=0.280). The AO fracture types were 31.3% C1, 50.0% C2, and 18.7% C3 in the fixed angle group, compared to 50.0% C1, 25.0% C2, and 25.0% C3 in the variable angle group (p=0.114). The average delay of surgery was 11.72±3.22 days for the fixed angle group and 10.41±3.69 days for the variable angle group (p=0.135) (Table I).

**Table I:** Baseline characteristics of the patients in both groups.

Characteristic	Fixed angle LCP (n=32)	Variable angle LCP (n=32)	p-value
	Number (%) / Mean±SD		
Age (in years)	38.81±9.60	34.13±7.46	0.120
Sex			
Male	28 (87.5)	28 (87.5)	1.000
Female	4 (12.5)	4 (12.5)	
Mechanism of Injury			
Road traffic accidents	28 (87.5)	32 (100.0)	0.039
Falls on outstretched hands	4 (12.5)	0	
Side Affected			
Dominant Hand	12 (37.5)	8 (25.0)	0.280
Non-Dominant Hand	20 (62.5)	24 (75.0)	
AO Fracture Type			
C1	10 (31.3)	16 (50.0)	0.114
C2	16 (50.0)	8 (25.0)	
C3	6 (18.7)	8 (25.0)	
Average Delay of Surgery from Date of Injury (days)	11.72+3.22	10.41±3.69	0.135

Regarding the Mayo wrist score, at 3 months, the total score was significantly higher in the variable angle group (63.12±11.81) compared to the fixed angle group (48.75±11.90, p=0.005). Notably, satisfaction scores were significantly higher in the variable angle group (18.13±4.78 vs. 11.88±4.03, p=0.002). At 6 months, the total score remained higher for the variable angle group (75.93±9.16 vs. 64.37±14.59, p=0.025), with significant differences in pain (19.69±3.86 vs. 15.94±4.55, p=0.041) and range of motion (18.75±5.00 vs. 14.38±4.03, p=0.011). At 1 year, the total score continued to favour the variable angle group (91.87±7.27 vs. 81.25±16.17, p=0.044), with significant differences in range of motion (23.13±4.03 vs. 19.38±6.29, p=0.029) (Table II).

**Table II:** Comparison of Mayo wrist score among patients in two groups.

Mayo wrist score	Fixed angle LCP (n=32)	Variable angle LCP (n=32)	p-value
	Mean±SD		
**Three (3) months**			
Total	48.75±11.90	63.12±11.81	0.005
Pain	12.50±5.16	15.31±4.27	0.095
Range of motion	13.75±2.88	15.63±5.12	0.111
Satisfaction	11.88±4.03	18.13±4.78	0.002
Grip strength	10.63±4.78	13.44±2.39	0.083
**Six (6) months**			
Total	64.37±14.59	75.93±9.16	0.025
Pain	15.94±4.55	19.69±3.86	0.041
Range of motion	14.38±4.03	18.75±5.00	0.011
Satisfaction	20.31±5.61	22.19±2.56	0.287
Grip strength	13.44±3.96	15.31±2.86	0.188
**One (1) year**			
Total	81.25±16.17	91.87±7.27	0.044
Pain	18.13±5.43	21.56±3.01	0.085
Range of motion	19.38±6.29	23.13±4.03	0.029
Satisfaction	22.81±4.46	23.75±2.23	0.485
Grip strength	20.63±5.12	23.44±3.52	0.144

Functional outcome, at 3 months, all patients in the fixed angle group were rated as poor, whereas in the variable angle group, 18.7% were rated as good, 25.0% as fair, and 56.3% as poor (p=0.011). At 6 months, the variable angle group had 18.7% rated as excellent, 25.0% as good, 50.0% as fair, and 6.3% as poor, while the fixed angle group had 12.5% good, 68.7% fair, and 18.8% poor (p=0.162). At 1 year, 68.7% of the variable angle group were rated as excellent compared to 37.5% of the fixed angle group; additionally, 31.3% of the variable angle group were rated as good, with no fair or poor ratings, while the fixed angle group had 37.5% good, 12.5% fair, and 12.5% poor (p=0.135) (Table III).

**Table III:** Comparison of functional outcome based on Mayo wrist score among patients in two groups.

Mayo wrist score	Fixed angle LCP (n=32)	Variable angle LCP	p-value
	Number (%)		
**Three (3) months**			
Excellent	0 (0.0)	0 (0.0)	0.011
Good	0 (0.0)	6 (18.7)	
Fair	0 (0.0)	8 (25.0)	
Poor	32 (100.0)	18 (56.3)	
**Six (6) months**			
Excellent	0 (0.0)	6 (18.7)	0.162
Good	4 (12.5)	8 (25.0)	
Fair	22 (68.7)	16 (50.0)	
Poor	6 (18.8)	2 (6.3)	
**One (1) year**			
Excellent	12 (37.5)	22 (68.7)	0.135
Good	12 (37.5)	10 (31.3)	
Fair	4 (12.5)	0 (0.0)	
Poor	4 (12.5)	0 (0.0)	

Radiological outcomes based on Lindstrom’s criteria showed that at 3 months, the variable angle group had a significantly greater volar tilt (6.6±1.03°) compared to the fixed angle group (5.63±1.01°, p<0.001), with no significant differences in radial height, radial inclination, or ulnar variance. At 6 months, the variable angle group again had a greater volar tilt (6.74±1.06° vs. 5.86±1.04°, p=0.001), while other measures remained similar between groups. At 1 year, the volar tilt was still greater in the variable angle group (6.86±1.10° vs. 6.05±1.07°, p=0.004), with no significant differences in other parameters (Table IV).

**Table IV:** Comparison of Lindstrom’s criteria score among patients in two groups.

Lindstrom’s criteria score	Fixed angle LCP (n=32)	Variable angle LCP (n=32) SD	p-value
	Mean±		
**Three (3) months**			
Radial height (mm)	8.71+0.81	8.92+0.92	0.336
Volar tilt (degrees)	5.63+1.01	6.6+1.03	<0.001
Radial inclination (degrees)	22.8+1.02	23.1+1.01	0.246
Ulnar variance (mm)	0.72+0.50	0.76+0.52	0.754
**Six (6) months**			
Radial height (mm)	8.82+0.92	9.05+0.93	0.323
Volar tilt (degrees)	5.86+1.04	6.74+1.06	0.001
Radial inclination (degrees)	22.83+1.01	23.11+1.08	0.289
Ulnar variance (mm)	0.71+0.61	0.72+0.59	0.781
**One (1) year**			
Radial height (mm)	8.94+0.93	9.02+0.94	0.414
Volar tilt (degrees)	6.05+1.07	6.86+1.10	0.004
Radial inclination (degrees)	22.96+1.03	23.23+1.02	0.303
Ulnar variance (mm)	0.74+0.63	0.71+0.58	0.691

Radiological outcomes at three months showed no patients rated as excellent or poor. However, 50% of the variable angle group were rated as good compared to 25% of the fixed angle group, and 75% of the fixed angle group were rated as fair compared to 50% of the variable angle group (p=0.144). At 6 months, both groups had 6.3% of patients rated as excellent. The fixed angle group had 62.5% rated as good compared to 50.0% in the variable angle group, and 31.2% rated as fair compared to 43.7% in the variable angle group (p=0.162). At 1 year, 43.7% of the variable angle group were rated as excellent compared to 31.2% of the fixed angle group. Both groups had 25.0% rated as fair, with no poor ratings in either group (p=0.717) (Table V).

**Table V:** Comparison of radiological outcome based on Lindstrom’s criteria score among patients in two groups.

Radiological outcome	Fixed angle LCP (n=32)	Variable angle LCP (n=32)	p-value
	P-value		
**Three (3) months**			
Excellent	0 (0.0)	0 (0.0)	0.144
Good	8 (25.0)	16 (50.0)	
Fair	24 (75.0)	16 (50.0)	
Poor	0 (0.0)	0 (0.0)	
Six (6) months			
Excellent	2 (6.3)	2 (6.3)	0.162
Good	20 (62.5)	16 (50.0)	
Fair	10 (31.2)	14 (43.7)	
Poor	0 (0.0)	0 (0.0)	
One (1) year			
Excellent	10 (31.2)	14 (43.7)	0.717
Good	14 (43.8)	10 (31.3)	
Fair	8 (25.0)	8 (25.0)	
Poor	0 (0.0)	0 (0.0)	

**Table VI:** Complications among patients in two groups.

Complications	Fixed angle LCP (n=32)	Variable angle LCP (n=32)	P-value
	Number (%)		
Residual pain	2 (6.3)	2 (6.3)	1.000
Implant impingement	2 (6.3)	2 (6.3)	1.000
Surgical site infection	0 (0)	2 (6.3)	0.150
Restricted Wrist movement	4 (12.5)	2 (6.3)	0.391

Complications were similar between the two groups. Residual pain was reported in 6.3% of patients in both the fixed angle LCP and variable angle LCP groups (p=1.000). Implant impingement occurred in 6.3% of patients in each group as well (p=1.000). Surgical site infection was observed in 6.3% of the variable angle group but not in the fixed angle group (p=0.150). Restricted wrist movement was more common in the fixed angle group (12.5%) compared to the variable angle group (6.3%), but this difference was not statistically significant (p=0.391).

## Discussion

In this prospective randomised control trial study, comparing functional and radiological outcomes, all the patients treated with volar plating had good to excellent functional and radiological outcomes either treated with variable or fixed angle locking compression plates.

The Mayo wrist score was utilized to assess functional outcomes at various time points post-operatively. Our results demonstrated that patients treated with variable angle locking compression plates exhibited significantly better functional outcomes compared to those treated with fixed angle plates at three months, six months, and one year postoperatively. These findings align with previous studies by Kitay *et al*, Kawasaki *et al*, Jung *et al*, Nishiwaki *et al*, Regar *et al*, and Chen *et al*, who also reported superior functional outcomes with variable angle plates^11-15^. The improved functional outcomes observed in our study can be attributed to the enhanced stability and anatomical reduction achieved with variable angle plates, allowing for improved range of motion and grip strength activities like Driving a two-wheeler and weight lifting^16^.

Nishiwaki *et al*, in a prospective randomized comparative study of 120 patients with unstable intra-articular fractures of distal radius treated with Variable angle and Fixed Angle Locking Plate reported no significant differences in Functional outcome between the groups at any follow-up time. Patients with intra-articular fractures can expect good functional and radiological outcomes with fixed-angle and variable-angle locking plates^13^.

Regar *et al*, in a prospective study of 156 patients with unstable Intra-articular Distal radius Fractures treated with a Variable and Fixed angle volar locking plate reported that patients treated with a variable angle volar plate show better results as compared to a fixed angle locking plate^14^.

Chen *et al*, in a study with 47 patients with Distal Radius Volar Rim fractures managed with Variable angle locking compression plate and Fixed angle locking compression plate reported improved Functional outcomes Improved Range of motion and decreased Flexor tendon irritation in VA-LCP as compared to FA-LCP^15^.

Radiological assessment based on Lindstrom’s criteria revealed comparable results between the two groups in terms of radial height, radial inclination, and ulnar variance at all time points. However, variable angle plates demonstrated significantly better volar tilt compared to fixed angle plates at three months and six months post-operatively. This finding is consistent with the biomechanical advantage offered by variable angle plates, which allow for more precise screw placement and improved volar tilt restoration. The importance of volar tilt restoration in achieving optimal functional outcomes has been emphasized in previous studies by Teunis *et al*, Yamazaki *et al*, Hammer *et al*, Koenig *et al*, and Khatri *et al*, highlighting the clinical significance of our findings^17-21^.

The incidence of complications was similar between the two groups, with no significant differences observed in residual pain, implant impingement, or restricted wrist movement. However, a higher rate of surgical site infection was observed in the variable angle group, although this difference was not statistically significant. These findings are consistent with previous literature, as reported by Chia *et al*, Harness *et al*, Bakker *et al*, O’Shaughnessy and Orbay *et al*, suggesting that the choice of plate type may not significantly influence complication rates^22-27^.

Primary internal fixation of distal radius fractures with variable angle screws of the Volar locking plate facilitates early mobilisation and hence return to activities with a good range of motions, especially rotations^27^. In some patients, there is a restriction of volar tilt post-operatively due to distal placement of plate in variable angle group to better purchase sub-chondral bone. In the Fixed angle group, additional Kirschner wire was required more commonly than in the variable group to fix the Radial styloid and to purchase sub-chondral fragments^28^. In the variable group, screw loosening was more common as compared to the fixed angle group may be due to surgeon’s errors like improper locking and/or directing more offset than 15°.

There were certain limitations in our study. The study was done at a single centre, which reduced external validity, and the comparison was done only with one type of implant, despite various types of plates being available in the market. Additionally, the one-year follow-up period was short to assess functional and radiological outcome comparisons. Intra-operative reduction and fracture type played a very important role in the outcome. As our study was a randomised control trial, certain intra-articular fractures that were highly comminuted may have required column-specific fixation for better reduction and outcomes. Further studies with larger sample sizes and longer follow-up periods are needed to assess the long-term clinical and radiological implications of variable angle locking plates over fixed angle locking plates.

## Conclusion

In conclusion, our study suggests that variable angle locking compression plates offer superior functional outcomes and improved volar tilt restoration compared to fixed angle plates upto first year of follow-up in the treatment of unstable distal end radius fractures. While both plate types demonstrated comparable radiological outcomes and complication rates, the clinical benefits associated with variable angle plates warrant consideration in the surgical management of these fractures. However, further research is warranted to confirm our findings and address the limitations of this study.
